# Autologous, allogeneic hematopoietic cell transplantation and CAR-T/NK therapy: what is their real importance in PTCL?

**DOI:** 10.3389/fonc.2023.1195759

**Published:** 2023-08-30

**Authors:** Samuel C. F. Couto, Ariel Kowes, Camila S. Aurabi, Theo G. M. Oliveira, Paulo Klinger, Vanderson Rocha

**Affiliations:** ^1^ Laboratory of Medical Investigation in Pathogenesis and Directed Therapy in Onco-Immuno-Hematology (LIM-31), Department of Hematology and Cell Therapy, Hospital das Clínicas HCFMUSP, Faculdade de Medicina, University of São Paulo, São Paulo, Brazil; ^2^ Fundação Pró-Sangue–Hemocentro de São Paulo, São Paulo, Brazil; ^3^ Instituto D’Or de Ensino e Pesquisa, São Paulo, Brazil

**Keywords:** hematopoietic cell transplantation, peripheral T-cell lymphoma, CAR-T cell, CAR-NK cell, immunotherapy

## Abstract

Peripheral T cell lymphoma (PTCL) is a rare and aggressive type of non-Hodgkin’s lymphoma that affects mature T cells. This type of cancer is characterized by the abnormal growth of T cells, which can accumulate in the lymph nodes, spleen, bone marrow, and other organs, leading to a variety of symptoms. PTCLs are often difficult to diagnose and treat, and they have a poorer prognosis than other types of lymphoma. However, recent advancements in treatment options, such as targeted therapies have shown promise in improving outcomes for patients with PTCL. Here, we discuss the use of autologous and allogeneic hematopoietic cell transplantation (HCT) as a treatment strategy for patients with PTCL, as well as the recent treatment approaches based on advanced cellular therapy. The current evidence for the use of HCT in PTCL is mainly derived from registry data, retrospective studies, and expert opinion, as randomized trials are limited due to the low incidence and histological heterogeneity of PTCL subtypes.

## 1 Introduction

Peripheral T-cell lymphomas encompass a biologically and clinically heterogeneous group of lymphoproliferative disorders derived from mature T-cells (post-thymic lymphocytes). Much less frequent than B-cell lymphomas, they represent about 10-15% of the non-Hodgkin lymphomas (NHL) in the Western hemisphere (1, 2). On the most recent World Health Organization Classification of Hematolymphoid Tumors (WHO-HAEM5), the mature T-cell and NK-cell neoplasms have been grouped into 9 families based on characteristics like cell of origin/differentiation state, clinical scenario, disease localization and cytomorphology (3). PTCL most common subtypes are, in order of incidence, PTCL-not otherwise specified (PTCL-NOS), angioimmunoblastic T-cell lymphoma (AITL), anaplastic large cell lymphoma (ALCL), anaplastic lymphoma kinase–positive (ALCL ALK-positive), anaplastic large cell lymphoma, anaplastic lymphoma kinase–negative (ALCL ALK-negative), and enteropathy-associated T-cell lymphoma (EATL), and each of them is characterized by unique genetic, molecular, histopathologic, and clinical features (2, 4). Overall, PTCL patients have dismal prognosis, and the currently available treatment strategies are still unsatisfactory for both front line and relapsed/refractory (R/R) settings. ALCL, ALK-positive subtype, might be an exception according to the International T-Cell Project, which reported a 5-year overall survival (OS) of 70% (5). Regimens derived from the existing protocols for B-cell non-Hodgkin lymphomas, like CHOP (cyclophosphamide, doxorubicin, vincristine, prednisone) and CHOP-like, are the ones used as first-line therapy and depending on the patient’s performance status and response to chemotherapy, consolidation with high-dose chemotherapy and hematopoietic cell transplantation (HCT) can improve the outcomes. Nevertheless, the risk of refractoriness or relapse is high for most patients (1). Due to the PTCL disorders heterogeneity and their low incidence, it is more difficult to conduct prospective studies, and for that reason most of the current evidence for the treatment comes from phase II trials, retrospective studies, and expert opinion. Therefore, PTCL still represents a therapeutic challenge. The aim of this study is to discuss the existing data and present a review over the role of autologous and allogeneic HCT for PTCL, and the role of more recent cellular therapy, such as chimeric antigen receptor-T and -NK cell (CAR-T and CAR-NK) in the treatment of these diseases. To comprehensively summarize the data, we created two tables incorporating patient characteristics, transplant settings, histology subtypes, conditioning regimens, and outcomes, extracted from diverse studies (**Tables 1**, **2**).

**Table 1:** Studies evaluating autologous HCT in PTCL as first-line treatment.

Study	Year	Patients (n)	Histology subtypes (most common/ALK+ ALCL)	Pre-transplant Response (CR/PR)	Conditioning, Regimen	TRM	Survival	Median follow-up
RETROSPECTIVE STUDIES								
Jantunen et al. (6)	2004	37	14 PTCL-NOS 14 ALCL 9 other (ALK+ ALCL included)	28 CR 2 PR	BEAC (22%) BEAM (15%)	4 (11%)	5-year: OS 54% PFS 44% (OS ALCL 85 *vs* 35% other subtypes)	24 months
Yam et al. (7)	2016	48 (20 autoHCT / 28 observation)	6 PTCL-NOS 6 AITL 8 other (ALK+ ALCL excluded)	20 CR	No description	–	3-year: OS 72% PFS 41%	26.4 months
PROSPECTIVE STUDIES								
Reimer et al. (8)	2009	83 (55 autoHCT)	32 PTCL-NOS 27 AITL 24 other (ALK+ ALCL excluded)	40 CR 15 PR	Cy/TBI	3 (3.6%)	3-year: OS 48%	33 months
Corradini et al. (9)	2006	62 (46 autoHCT)	28 PTCL-NOS 19 ALK+ ALCL 15 other (ALK+ ALCL included)	32 CR 10 PR	Mitoxantrone + Melphalan BEAM	3 (4.8%)	12-year: OS 34% DFS 55% EFS 30%	76 months
Rodríguez et al. (10)	2007	26 (19 autoHCT)	11 PTCL-NOS 8 ALK+ ALCL 7 AITL (ALK+ ALCL included)	17 CR 2 PR	BEAM	–	3-year: OS 73% PFS 53%	35 months
Mercadal et al. (11)	2008	41 (17 autoHCT)	20 PTCL-NOS 12 AITL 9 other (ALK+ ALCL excluded)	20 CR 4 PR	BEAM BEAC	1 (2.4%)	4-year: OS 39%	3.2 years
D’Amore et al. (12)	2012	166 (115 autoHCT)	62 PTCL-NOS 31 ALK- ALCL 30 AITL 21 EATL 16 other (ALK+ ALCL excluded)	82 CR 49 PR	BEAM BEAC	7 (4%)	5-year: OS 51% PFS 44%	60.5 months
Wilhelm et al. (13)	2016	111 (75 autoHCT)	42 PTCL-NOS 37 AITL 16 ALK- ALCL 16 other (ALK+ ALCL included)	69 CR 22 PR	Cy/TBI BEAM	(3,6%)	5-year: OS 44%, DFS 54% PFS 39%	59 months
Registry studies Rodríguez et al. (14)	2003	115	72 PTCL-NOS 25 ALCL 18 other (ALK+ ALCL included)	37 CR 28 CR2 44 PR	BEAM (43%) BEAC (32%) Cy/TBI (12%) Other (12%)	9 (8%)	5-year: OS 56% DFS 60%	37 months
Park et al. (15)	2019	119 (36 autoHCT)	**- autoHCT** 17 AITL 15 PTCL-NOS 4 ALK- ALCL **- non-autoHCT** 18 AITL 39 PTCL-NOS 26 ALK- ALCL	CR1	BEAM BEAM variation	–	2-year: **- autoHCT** OS 87.8% (95% CI, 77.3%-99.8%) PFS 57.6 (*P*=0.23) **- non-autoHCT** OS 70.2% (95% CI, 60.9%-80.9%) PFS 47.5%	2.8 years
Al-Mansour et al.	2019	28 (15 autoHCT, 3 did not undergo transplant)	11 PTCL-NOS 10 ALCL 7 AITL (ALK+ ALCL included)	CR/PR	CBV TBI + Etoposide/Cy	–	5-year: **- autoHCT** OS 40% PFS 40% **- non-autoHCT** OS 45% PFS 38%	7.8 years

PTCL-NO, Peripheral T-cell Lymphoma, not Otherwise Specified; ALCL, Anaplastic Large Cell Lymphoma; AITL, Angioimmunoblastic T-Cell Lymphoma, CR, Complete Response; PR, Partial Response; CR1, First Complete Remission; CR2, Second Complete Remission; OS, Overall survival; PFS, Progression-Free Survival; EFS, Event-Free Survival; DFS, Disease-Free Survival; Cy, Cyclophosphamide; TBI, Total Body Irradiation; Beam (Carmustine, etoposide, citarabyne, melphalan); BEAC (Bleomycin, etoposide, doxorubicin, cyclophosphamide; CBV (Carmustine, etoposide, Cyclophosphamide).

**Table 2:** Studies evaluating autologous and allogeneic HCT in R/R PTCL.

Study	Type of transplant	Patients (n)	Histology subtypes	Disease status at HCT	Conditioning, regimen	TRM	Outcomes	Median follow-up
RETROSPECTIVE STUDIES								
Chen et al. (16)	Autologous	53	18 ALCL 16 Unspecified 9 AITL 7 nNK/T 2 HSTL 1 ATLL	15 CR1/PR1 28 CR2/PR2+ 10 Refractory	CR1/PR1: 13 chemotherapy only 2 TBI-based CR2/PR2+: 24 chemotherapy only 4 TBI-based Refractory: 7 chemotherapy only 3 TBI-based	4% NRM	5-year PFS: CR1/PR1 51% CR2/PR2+ 12% Refractory 0 5-year OS: CR1/PR1 76% CR2/PR2 40% Refractory 30%	60 months
Rodriguez et al. (14)	Autologous	115	72 PTCL-NOS 25 ALCL 8 Lymphoepiteloid 6 AITL 3 HSTL 1 EATL	37 CR1 28 CR2+ 44 PR 6 Refractory	50 BEAM 37 BEAC 14 Cy-TBI 10 CVB 4 Others	8% TRM	5-year OS: CR1 80% CR2+ 50% PR1+ 49% Refractory 0	37 months
Huang et al. (17)	Autologous Allogeneic	67 (43 autologous, 24 allogeneic)	Autologous: 20 PTCL-NOS 18 ALCL, ALK- 5 nNK/T Allogeneic: 17 PTCL-NOS 1 ALCL, ALK- 1 AITL 5 nNK/T	Autologous: 20 CR1 6 CR2 7 PR 10 Refractory Allogeneic: 0 CR1 2 CR2 6 PR 16 Refractory	Autologous: 38 BEAM 5 Others Allogeneic: 6 Cy-TBI 18 Bu-Cy	Autologous: 1-year NRM 7% Allogeneic: 1-year NRM 18%	Autologous: Median time from HCT to relapse: 6 months 5-year PFS 49% 5-year OS 59% 3-year PFS 20% (primary refractory specifically) 3 year OS 20% (primary refractory specifically) Allogeneic: Median time from HCT to relapse: 8 months 5-year PFS 54% 5-year OS 55% 3-year PFS 49% (primary refractory specifically) 3 year OS 53% (primary refractory specifically)	Autologous: 31 months Allogeneic: 25.5 months
Rohlfing et al. (18)	Autologous Allogeneic	117 (89 R/R)	34 PTCL-NOS 31 ALCL,ALK- 28 AITL 11 nNK/T 10 EATL 3 HSTL	No description	Autologous: Dexa-BEAM Allogeneic: 18 Myeloablative 12 RIC 1 Unknown	Autologous: 0 Allogeneic: 23% TRM	Autologous (n=7): Median survival 10 months Death from PD 100% Allogeneic (n=31): Median survival not reached 5-year OS 52% No transplant (n=51): Median survival 3 months Death from PD 92%	5.8 years
Smith et al. (19)	Autologous Allogeneic	241 (115 autologous, 126 allogeneic)	Autologous: 61 ALCL (ALK+, - and unknown) 39 PTCL-NOS 15 AITL Allogeneic: 51 ALCL (ALK+, - and unknown) 63 PTCL-NOS 12 AITL	Autologous: 40 CR1 24 CR2+ 16 PR1 17 PR2+ 16 Refractory 2 no data Allogeneic: 18 CR1 20 CR2+ 23 PR1 21 PR2+ 41 Refractory 3 no data	Autologous: 26 TBI-based 65 BEAM 14 Cy 4 Bu-Mel / Bu-Cy 6 Other Allogeneic: 74 Myeloablative 45 NST/RIC 7 Unknown	Autologous: 6% NRM Allogeneic: 34% NRM	Autologous: 3-year PFS 42% 3-year OS 53% (excluded CR1) Allogeneic: 3-year PFS 31% 3-year OS 41% (excluded CR1)	48 months
Czajczynska et al. (20)	Allogeneic	24	9 PTCL-NOS 5 AITL 4 ALCL (1 positive / 3 negative) 2 EATL 2 nNK/T 1 T-PLL 1 LTCL	2 CR1 5 CR2 2 CR2+ 6 PR1 4 PR2 1 SD 1 Refractory 1 Resistance relapse 2 Responding relapse	21 BEAM-Alemtuzumab 3 Other	25%	100-day OS 87.5% 1-year OS 58.3% 3-year OS 42.4%	44.8 months
Wulf et al., 2019 (21)	Allogeneic	84	30 PTCL-NOS 17 AITL 15 ALCL 4 nNK/T 5 LTCL 6 T-PLL 7 Other	13 CR 35 PR 14 SD 22 PD/Refractory	FBC-12 (Myeloablative)	13.1% at 1 year 32.3% at 3 years 46% at 5 years	38.2% OS at median follow-up 37.2% DFS at median follow-up	14.5 months
PROSPECTIVE STUDIES								
Shustov et al. (22)	Allogeneic	17 (14 R/R)	7 PTCL-NOS 4 AITL 3 T-PLL 1 ALCL 2 Other	8 CR 5 PR 2 SD 2 PD	Flu-TBI (non myeloablative)	3-year NRM 19%	3-year OS 59% 3-year PFS 53%	3.3 years
Jacobsen et al. (23)	Allogeneic	52	20 PTCL-NOS 6 ALCL 5 AITL 4 HSTL 4 nNK/T 1 ATLL 1 EATL 9 Other	10 CR1 7 CR2 6 CR3 16 PR 5 Relapse	31 Myeloablative 21 RIC	3-year NRM: 36% Myeloablative 14% RIC	3-year OS 41% 3-year PFS 30% Relapse at 3 years: Myeloablative 33% RIC 57%	49 months
Corradini et al., 2004 (24)	Allogeneic	17	9 PTCL-NOS 4 AITL 4 ALCL ALK-	1 CR2 12 PR2+ 1 CR3 2 PD 1 untested	RIC	2-year NRM 6%	3-year OS 81% 3-year PFS 64%	28 months

AITL, angioimmunoblastic T-cell lymphoma; ALCL, anaplastic large cell lymphoma; ATLL, adult T-cell leukemia/lymphoma; BEAC, bleomycin, etoposide, doxorubicin and cyclophosphamide; BEAM, carmustine, etoposide, cytarabine and melphalan; Bu-Cy, busulfan-cyclophosphamide; Bu-Mel, busulfan-melphalan; CR1/PR1, first complete or partial response; CR2/PR2+, second or more complete or partial response; CVB, cyclophosphamide, etoposide, BCNU; Cy-TBI, cyclophosphamide-TBI; Dexa-BEAM, dexamethasone-BEAM; DFS, disease-free survival; EATL, enteropathy-associated T-cell lymphoma; FBC-12, fludarabine, busulfan and cyclophosphamide; Flu, fludarabine; HCT, hematopoietic cell transplantation; HSTL, hepatosplenic T-cell lymphoma; LTCL, Lymphoblastic T cell lymphoma; nNK/T, nasal type extranodal NK/T cell lymphoma; NRM, nonrelapse mortality; NST, nonmyeloablative; OS, overall survival; PD, progressive disease; PFS, progression free survival; PR, partial response; PTCL, peripheral T-cell lymphoma; PTCL-NOS, PTCL-not otherwise specified; RIC, reduced intensity conditioning; R/R, relapsed/refractory; SD, stable disease; TBI, total body irradiation; T-PLL, T-cell prolymphocytic leukemia; TRM, treatment related mortality.

## 2 First-line HCT in PTCL

The first nation-wide survey conducted in transplanted PTCL patients was in Finland during 1990-2001. Following induction therapy, patients were submitted to high dose therapy (HDT) conditioning regimen before autologous HCT (autoHCT). Of thirty-seven patients assessed, four (11%) died from treatment related mortality (TRM), 76% (28/37) achieved complete response (CR) and 5% (2/37) were in partial remission (PR). Eight percent of patients (3/37) were refractory to HDT and died from progressive lymphoma. The 5-year OS was 63% after autoHCT in frontline CR/PR vs 45% beyond second treatment. This study included fourteen patients (37%) with ALCL, and these patients had a significantly better OS compared to other PTCL subtypes (85% vs 35%). Although relatively high TRM rate (11%), this nation-wide survey supports the idea that HDT followed by autoHCT is feasible and that higher survival rates correlates with response to treatment and with specific PTCL subtypes. However, prospective randomized trials are needed to better determine the impact in up-front PTCL therapy (6). Rodriguez et al. described the GEL-TAMO (Grupo Español de Linfomas/Trasplante Autólogo de Médula Ósea) experience in patients with PTCL who underwent HDT and autoHCT. One hundred and fifteen patients were included and given mostly anthracycline-based regimens prior to transplant. Complete response was achieved in 86% of patients (98/114). Six patients (5%) achieved PR, 3% (3/114) had stable disease and 6% (7/114) disease progression. At a median follow-up of 37 months, 73 (64%) patients were alive with an estimated 5-year OS of 56% and disease-free survival (DFS) of 60%. Forty-two patients (37%) had died, with the main cause being disease progression in 32 of these patients (76%). The 5-year OS in first-line CR was significantly higher in comparison to second-line or more CR prior to transplant (80% and 50%, respectively). Data from this study shows a higher survivability in chemosensitive PTCL patients. Prognostic criteria, like adjusted-IPI (a-IPI) higher than 1 and altered LDH were associated with lower 5-year OS (a-IPI 0-1: 65% vs a-IPI>1: 13%; LDH normal 55% vs high LDH: 22%) in univariate analysis and therefore, might have utility in predicting clinical outcomes (14).

A meta-analysis published in 2016 by El-Asmar et al. (25) collected and analyzed data from 27 studies (3 prospectives and 16 retrospectives for front-line) and assessed the efficacy of HDT with autoHCT in front-line and R/R consolidation in PTCL. The pooled prospective and retrospective trials included a total of 179 and 599 patients, respectively. Interestingly, as expected, there was a clear difference between the OS and progression-free survival (PFS) on the different types of studies, with OS rates of 54% and PFS 33% in prospective, 68% and 55% in the retrospective studies, respectively. TRM pooled rates in prospective were 2% versus 6% in retrospective studies. These results are probably due to better control of confounding factors and accurately defining death causality in prospective trials. Although with several limitations in this study and the lack of randomized trials, the meta-analysis allowed strong evidence to support HDT and autoHCT as a reasonable option in front-line treatment for PTCL (25).

Moreover, Yam and colleagues compared PFS in first complete response (CR1) PTCL patients submitted to autoHCT or active observation after CHOP-like regimens. In a total of 48 patients (28 in the observation group and 20 submitted to consolidation transplant), the median follow-up duration was 26.4 months. The median PFS was 15.8 months for the observation patients and 12.8 months for patients who underwent autoHCT. The estimated 3-year PFS was 37% and 41% for the observation and transplantation groups, respectively. The results of this study revealed no improvement in PFS and OS in patients who achieved CR1 with observation or autoHCT. Even though these findings have several limitations, including the nature of the study, it shows the diversified data between different trials in PTCL patients, and prospective randomized trials could provide stronger evidence in this matter (7).

A prospective PTCL-restricted multicenter study that evaluated the role of frontline therapy with myeloablative chemoradiotherapy (CRT) and autoHCT was conducted by Reimer et al. From June 2000 to April 2006, a group of 83 patients with PTCL were given four to six cycles of CHOP, and if at least PR was reached, they proceeded to mobilization and were submitted to CRT followed by autoHCT. After CHOP therapy, patients had an overall response rate (ORR) of 79% (39% CR and 40% PR), and 66% (55/83) patients completed myeloablative therapy and proceeded to autoHCT. The main reason for not undergoing transplant was disease progression (22 patients). Following transplantation, 48 out of 55 patients achieved CR and seven patients achieved PR. In the intention to treat analysis, ORR was 66% (58% CR and 8% PR). After a median follow-up of 33 months, 43 patients (52%) were still alive either in remission (35) or with evidence of disease (8). The estimated 3-year OS rate was 48%. The estimated 3-year OS rate was 71% for patients who underwent autoHCT compared with only 11% for patients who did not undergo autoHCT. These findings, although with limitations, suggest a favorable outcome in autoHCT compared with conventional chemotherapy alone (8).

Corradini et al. reported a median 76-month follow-up of 62 patients with PTCL in Italy healthcare institutions. Patients were submitted to high-dose sequential chemotherapy regimen followed by autoHCT. Prior to the autologous transplant, 56% (32/62) patients were in CR, 10 (16%) were in PR and 15 patients (24%) had progressive disease (PD). The intention-to-treat analysis showed that forty-six out of the sixty-two (74%) of patients underwent autologous transplantation, whereas sixteen patients did not undergo transplant, mainly due to disease progression. After autoHCT, 89% (41/46) patients were in CR, with 5 patients (11%) in PR that died shortly after disease progression. At a median follow-up of 66 months, the estimated 12-year OS, DFS and event-free survival (EFS) of 34%, 55% and 30%, respectively. Due to inclusion of ALK-positive ALCL (31%), with overall better prognosis, these results should be interpreted with caution. Multivariate analysis showed that patients with CR before autoHCT had a statistically significant benefit in terms of OS and EFS. These findings suggest that achieving CR before autoHCT could offer a greater chance of long-term survival (9).

The GEL-TAMO Study Group published a phase II trial for front-line treatment for high-risk nodal PTCL with MegaCHOP (higher-dose CHOP) regimen and consolidation with autoHCT. Twenty-six patients were enrolled to the study, and after the first three courses of MegaCHOP, response was evaluated with computed tomography (CT) and gallium scans. In case of CR and negative gallium scan, patients were assigned to receive one to two additional courses of MegaCHOP and the conditioning regimen followed by autoHCT. The remaining patients were given salvage therapy with IFE (ifosfamide, etoposide) and those who achieved at least PR in re-evaluation would proceed to autoHCT. The remaining patients were considered as primary failure and were excluded from the protocol. After a median follow-up of 35 months after diagnosis, the 3-year OS and PFS was 73% and 53%, respectively. In addition, 73% (19/26) of patients who received autoHCT presented an estimated 2-year OS, PFS and DFS of 84%, 56% and 63%, respectively. The only variable in this study that showed significant impact on OS was the chemosensitive status, both after initial MegaCHOP and before transplant, with a 3-year OS of 83% for patients with complete or partial response after three courses of MegaCHOP, compared to 43% OS in patients with primary refractory disease. Even patients that were chemosensitive after IFE salvage therapy appeared to have an improvement in the estimated 3-year OS. Univariate analysis of prognostic score systems, like Prognostic Index for PTCL (PIT) and a-IPI, showed no difference in outcome with this treatment strategy. They concluded that frontline autoHCT might have overcome the poor prognosis determined by prognostic scoring systems. The approach of salvage therapy in high-risk aggressive nodal PTCL that does not achieve CR in initial treatment may improve outcomes following autoHCT (10).

Mercadal et al. studied 41 patients with newly diagnosed PTCL and submitted them to either MegaCHOP or ESHAP (etoposide, cisplatin, cytarabine and prednisone) regimen, with consolidation autoHCT if at least PR was reached. Seventeen patients failed therapy, with sixteen cases because of disease progression and one due to an early death by severe infection. Twenty-four patients (16 CR, 4 CR/unconfirmed (CRu) and 4 PR) were candidates for autoHCT. The authors discuss an important selection bias in studies aimed at evaluating the role of autoHCT, mainly because a significant proportion of patients do not respond to induction chemotherapy and therefore, are not submitted to transplantation. Within this study, among the 58% patients eligible for transplantation, only 41% eventually received a transplant out of the initial. These results might be due to a moderate CR rate obtained and the small sample size, and novel therapies and clinical trials should be encouraged to improve outcomes in PTCL therapy (11).

The Nordic Lymphoma Group (NLG) conducted one of the largest prospective phase II studies that addressed the role of up-front HDT and autoHCT in PTCL. With a total of 166 patients enrolled, treatment consisted of CHOEP-14 (CHOP with inclusion of etoposide biweekly), or CHOP-14 (CHOP biweekly) in patients aged over 60 years. Patients who achieved PR or CR were candidates for conditioning chemotherapy and autoHCT. Patients were evaluated for treatment response, and at the end of induction treatment, 82 of 156 patients achieved CRu and 49 PR, with an ORR of 82%. Twenty-five patients developed primary refractory disease. For various reasons, 16 patients were excluded before HDT and autoHCT, finally resulting in 115 patients who continued to transplantation. With a median follow-up of 60.5 months, the 5-year OS and PFS was 51% and 44%, respectively. Mortality due to treatment toxicity resulted in seven deaths, corresponding to a 4% TRM. ALCL ALK-positive, primary cutaneous, and primary leukemic subtypes were excluded. The highest OS and PFS after subtype-specific analysis were seen in patients with ALCL ALK-negative (5-year OS 70% and PFS 61%). The median 5-year follow-up allowed analysis of the time to disease relapse, with 11 patients (7%) developing relapsed disease after two years from transplantation, and therefore, provide rationale for a future maintenance therapy. Almost one fourth of patients (26%) failed treatment prior to transplant, reinforcing the need for better and novel therapies in the first-line treatment for PTCL. The results of this large cohort revealed encouraging outcomes and strengthened the recommendation of HDT and autoHCT for first-line therapy in PTCL patients (12).

The German study from Willhelm et al. reported a median 5-year follow-up study with 111 patients with newly diagnosed PTCL. Patients received conventional chemotherapy regimen with CHOP and if at least PR was reached, patients would proceed to myeloablative therapy and autoHCT. Treatment response revealed 91 (82%) ORR, with 69 patients (62%) achieving CR and 22 a PR (20%), with the remaining 20 patients (18%) failing primary treatment. Only 75 (68%) completed the entire study protocol, with disease progression/relapse as the main cause for not undergoing autoHCT. The estimated 5-year OS, DFS and PFS were 44%, 54% and 39% respectively. Considering patients who underwent autoHCT, OS rate was estimated at 57%, in comparison to 23% OS in those who did not. TRM rate resulted in 3.6%, similar to previous prospective studies. Long follow-up analysis showed that 43 (39%) patients achieved continuous remission, but early relapse and disease progression remained as major issues and demand novel treatment strategies to improve response rates in PTCL, allowing more patients to proceed with transplantation (13).

A registry study from Park et al. analyzed data from the prospective multicenter cohort study COMPLETE (Comprehensive Oncology Measures for Peripheral T-Cell Lymphoma Treatment), analyzed the impact of autoHCT on outcomes of patients with nodal PTCL in first-line complete remission. This was the first published study to report findings from prospective enrolled patients comparing clinical outcomes with or without autoHCT. Of the 119 patients with nodal PTCL who achieved CR1, 36 (30%) underwent autoHCT, while 83 (70%) were treated without autoHCT. Physician choice was the main reason for not considering transplantation, accounting for 55% of cases, which could serve a potential bias, as higher proportions of AITL histology subtype, advanced-stage disease (III/IV), and patients younger than 65 years were reported in the autoHCT group. ALCL subtype, which is associated with a favorable prognosis, was more frequently present in the non-autoHCT group. Overall, after a median follow-up of 2,8 years, there was no significant difference in survival between patients with CR1 that underwent and who did not undergo autoHCT. However, subgroup analysis suggested better outcomes in patients with AITL that proceeded to autoHCT compared to other nodal PTCL subtypes, with improved outcomes in OS in advanced-stage disease and intermediate to high IPI. The estimated 2-year OS and PFS rates for CR1 patients were 75.3% and 63.4%, respectively; while patients who did not achieve CR after first-line chemotherapy was 41.9% and 19.3%, respectively. Although with a notable selection bias limitation, these findings strengthen the importance of achieving CR1 in OS and serve as guidance for new and larger studies to determine the real benefit of autoHCT in PTCL (15).

Rarer entities such as hepatosplenic T-cell lymphoma (HSTL), AITL and EATL are underrepresented in clinical trials, making it difficult to find data regarding treatment approaches and outcomes in a real-world setting. As described in registry studies and other retrospective and prospective studies, consolidative autoHCT should be considered for AITL cases in first CR (7, 8, 10–13). The supporting evidence for utilizing autoHCT as a consolidation tactic is most compelling for EATL. One study conducted by the Scotland and Newcastle Lymphoma Group involved 26 patients who underwent a unique treatment regime that consisted of one round of CHOP followed by three rounds of IVE (ifosfamide, vincristine, etoposide), with alternating intermediate-dose methotrexate. Subsequently, autoHCT was administered if the patients were in remission. The 5-year PFS and OS rates were found to be 52% and 60% respectively, which displayed significant improvement when compared to the historical group treated with anthracycline-based chemotherapy (26). In a UK phase II study, 21 patients (including 11 with EATL) were assessed using the induction regimen applied by the Scotland and Newcastle Lymphoma Group. For EATL patients, both the 1-year OS and PFS rates were 45%. Among the five EATL patients who underwent autoHCT, only one experienced relapse (27). Furthermore, the already presented NLG study had 21 EATL patients enlisted, demonstrating a 5-year PFS rate of 38% and an OS rate of 48% for this specific group of patients (12). Supporting these findings, a retrospective analysis of the European Society for Bone and Marrow Transplantation (EBMT) registry and a retrospective cohort study both highlighted a survival advantage with autoHCT (28, 29). To date, from the limited data available for HSTL, it appears that the best therapeutic approach is non-CHOP induction therapy followed by consolidation with allo or auto (if limited donor availability) HCT (30, 31).

The SWOG S9704 trial designed by lymphoma committees of the United States and Canada was the first randomized trial to address consolidative autoHCT for high-risk patients with diffuse aggressive NHL. After patients received five cycles of induction chemotherapy with CHOP or R-CHOP (CHOP plus rituximab), they were randomized either to receive three more cycles of chemotherapy (control group) versus one additional cycle followed by autoHCT with prior myeloablative radio or chemotherapy-based regimen. In a total of 370 eligible patients, 40 had an aggressive T-cell phenotype NHL. Of these, 28 (70%) patients were randomized after induction therapy, with 9 out of 12 patients excluded due to early disease progression. Thirteen were in the control arm and 15 in the transplantation arm, with three patients that did not undergo transplant due to patient refusal (2) or mobilization failure (1). At a median follow-up of 7.8 years after randomization, there was no statistical significance observed in the 5-year estimated OS (40% versus 45%) and PFS (40% versus 38%) for the transplant and control group, respectively. While results were discouraging for the first randomized trial, these findings should be analyzed with caution due to the small sample size and the retrospective analysis of a specific subgroup from the study (32).

Savage et al. reported a subgroup analysis of the role of HCT in CD30+ PTCL in the double-blind randomized phase III ECHELON-2 study after frontline Brentuximab vedotin (BV) plus cyclophosphamide doxorubicin and prednisone (CHP) versus CHOP regimen. From the BV plus CHP arm, 114 of 177 (64%) patients were in CR at the end of the treatment regimen. Thirty-eight of 114 (33%) underwent consolidative HCT (2 allo and 36 auto). With a median follow-up of 47.57 months, there was no difference in adverse event profile for those who did or did not undergo transplantation, with an estimated 3-year PFS of 80.4% vs 54.9%, respectively. The estimated 5-year PFS was 65.3% after transplant vs 46.4% without transplant. Of the CHOP arm, 97 of 177 (55%) were in CR at end of treatment. Twenty-nine out of 97 patients underwent consolidative transplant. At a median follow-up of 53.72 months, the estimated 3-year PFS was 67,2% in favor for consolidative transplant vs 54.1% in those who did not undergo transplant. The results for 5-year PFS were 48.9% and 40.9%, respectively. Even though the ECHELON-2 trial was not focused on transplantation, analysis of HCT seems to support a benefit in consolidative HCT for patients that received BV plus CHP, with less pronounced benefit in the CHOP arm. The low sample size of the study limits the statistical power and the overall impact of consolidative HCT (33).

The French Lymphoma Study Association (LYSA) and the German Lymphoma Alliance (GLA) designed a prospective, randomized, multicenter, phase III trial that evaluated the role of allogeneic HCT (alloHCT) against autoHCT in untreated patients with high-risk PTCL. Patients were randomized to either receive four courses of CHOEP-14, one course of DHAP (dexamethasone, cytosine-arabinoside, and cisplatin or carboplatin) and auto or alloHCT after conditioning regimen. From 103 patients in the intention-to-treat analysis, 54 were assigned to autoHCT and 49 to alloHCT. Thirty-four of 54 patients (63%) underwent autoHCT. Twenty patients were unable to proceed to transplant, 15 due to early progression. Twenty-six of 49 patients (53%) underwent alloHCT, while 14 patients did not undergo transplant due to early progression. Eight patients randomized to alloHCT had no compatible donor and were rescheduled to receive autoHCT. In total, 41 patients were consolidated with autologous HCT and 26 with alloHCT. At a median follow-up of 42 months, there were no significant differences between auto and alloHCT in OS (70% vs 57%), PFS (39% vs 43%) and EFS (38% vs 43%), respectively. TRM rate was 0% in the autoHCT setting, while eight deaths (31% TRM) were related to alloHCT. Almost a third of patients did not undergo transplant due to disease progression, supporting the rationale for more effective and novel therapies for T-cell lymphoma. The significant high TRM observed in first-line alloHCT is not acceptable in current treatment settings, and therefore, the recommendation for alloHCT should be mainly in specific histologic subtypes or patients with R/R disease, while autoHCT continues to be the preferred option for patients with newly diagnosed PTCL (34).

Given the conflicting data, small sample size, numerous histology subtypes and different treatment regimens, the impact of consolidation therapy with autologous HCT in patients with PTCL is controversial. Many retrospective and prospective studies tend to suggest benefits for autoHCT in up-front therapy. Current guidelines and expert opinion recommend this strategy as the main treatment choice in most of the more common subtypes, excluding ALCL ALK-positive, extranodal NK/T-cell lymphoma and Adult T-cell Leukemia/Lymphoma (ATLL) (2, 35). Treatment related toxicity is generally manageable, showing feasibility of chemotherapy and autoHCT in newly diagnosed patients. The predictive capability of prognostic scoring systems prior to transplant might be surpassed with this first-line treatment strategy, although still debatable. The value of achieving the best ORR, mainly CR, is crucial for improving clinical outcomes and OS and PFS, and therefore, up-front autologous HCT might benefit with a long-term disease remission in chemosensitive patients. With novel and promising therapies emerging, defining the real role for autologous HCT in PTCL frontline therapy is still challenging, and larger and transplant-focused randomized trials are required to establish it.

## 3 HCT in R/R PTCL

Unfortunately, disease refractoriness and relapse are common outcomes for patients with peripheral T-cell lymphoma. Data collected from patients enrolled in the International T-cell Project from 2006 to 2016 showed that out of the patients that received first line therapy, 32% reached and sustained CR and 68% were refractory or relapsed. Among those labeled as refractory/relapsed, 69% represented the refractory and 31% the relapsed (1). Median time from diagnosis to relapse ranges from 8 to 12.1 months (1, 36). Regarding survival rates, the COMPLETE Registry showed that the median OS were 29.1 months for relapsed patients and 12.3 months for refractory patients, which suggests that patients with chemosensitive disease have higher survival rates. They also found that 30% of this population had T-cell lymphomas with extra nodal involvement (36). In the International T-cell Project, after a median follow up of 38 months, 70% of the R/R patients had died, and the median survival time after relapse was only 5.8 months. Three-year OS were 21% and 28% for the refractory and relapsed, respectively. They also demonstrated that refractory disease was associated with higher risk of death, while later relapse (> 12 months) and salvage therapy with stem cell transplant were associated with better OS (1).

A retrospective study from Stanford University showed that disease status by the time of transplant had a great impact on OS and PFS. Fifty-three patients with PTCL underwent autoHCT in different stages of disease. Five-year PFS rates were 51%, 12% and 0 for patients in CR1/PR1, CR2/PR2 and primary refractory disease, respectively. Corresponding 5-year OS rates were 76%, 40% and 30% (16). Similarly, the GEL-TAMO presented data from 115 PTCL patients treated with autologous HCT in CR1, CR2+, PR1+ or refractory disease, and they also found higher survival rates in patients who were transplanted in CR1 in comparison to other groups. For patients in CR1, CR2+, PR1+ and refractory disease, the 5-year OS were 80%, 50%, 49% and 0, respectively. They also concluded that transplant in first line or chemosensitive disease, age < 41 years old, ECOG 0 or 1, absence of extranodal involvement, among other factors, are associated with higher survival (14). Both studies show that autoHCT performed in earlier stages of remission was associated with better outcomes, highlighting those patients with refractory disease had much worse performance, and those relapsed with chemosensitive disease were more likely to benefit from this strategy.

A systematic review and meta-analysis evaluated HDT and autoHCT for PTCL. They included 27 studies, in which 15 reported autoHCT in the R/R PTCL setting. PFS, OS, relapse/progression and TRM pooled rates were 36%, 47%, 51% and 10%, respectively. These data point to HDT/autoHCT as a reasonable strategy for R/R PTCL, given its 47% OS rate, but it also represented higher TRM when compared to HDT/autoHCT in first-line. An important discussion raised by these authors is the fact that most studies assess patients with relapsed or refractory disease combined, and therefore the outcomes for them seem to be the same, however, they believe that patients with refractory disease may not achieve the same outcomes than those that have relapsed disease, but have previously presented some chemosensitivity to salvage therapy, since this is a predictor of response to HDT/auto-HCT in other types of lymphoma (25).

Allogeneic HCT data for PTCL is mostly based on retrospective and prospective single-arm studies. However, the recommended treatment strategy in the R/R setting of non-ALK+ PTCL is salvage chemotherapy followed by HCT (autoHCT or alloHCT) (37). For patients with primary refractory PTCL, or PTCL that has relapsed after autoHCT or multiple prior lines of therapy, alloHCT provides the only potential curative therapy with survival rates of 40 to 50%. Due to the high risk of NRM, particularly with myeloablative conditioning in patients who have recently received an autograft or who have received extensive salvage chemotherapy, reduced intensity regimens are preferred (19–23, 38, 39).

AITL presents a rather unique scenario where alloHCT in the R/R setting appears to hold more promise compared to other nodal PTCLs. According to data from the CIBMTR, R/R AITL patients demonstrated a 4-year PFS and OS of 47% and 56%, respectively. Notably, relapse rates maintained a steady level at the 2-year mark post alloHCT, indicating sustained disease control even in patients who had experienced a failed prior autoHCT and those with refractory disease at the time of alloHCT (4-year PFS: 38%, OS: 52%) (40). Furthermore, the French registry data showed a favorable survival advantage for R/R AITL when compared to other histological subtypes (PTCL and ALCL), showing 5-year OS and EFS rates of 80% for R/R AITL. Despite the differences in survival rates, the univariate analysis did not reveal any statistical significance in OS, EFS, and TRM among the various histological subtypes (41).

A retrospective study from Huang et al. comparing auto and alloHCT for PTCL showed that primary refractory patients that underwent autoHCT had 3-year PFS of 20% and 3-year OS of 20%, while alloHCT provided rates of 49% and 53% for 3-year PFS and 3-year OS, respectively (17). Additionally, in a study aimed at evaluating salvage strategies after relapse in PTCL ALK-, all seven patients that underwent autoHCT at relapse died from disease progression, with median survival of 10 months (18). On the other hand, Smith et al. described a cohort of 241 patients from the Centre for International Blood and Marrow Transplant Research (CIBMTR) database that showed better OS and PFS in patients that underwent autoHCT in comparison to alloHCT, although there were differences in baseline characteristics (patients from the autologous group were more likely to be in CR1, have chemosensitive disease, ALCL subtype and fewer previous lines of therapy, which means they were of lower risk than the patients from the allogeneic group). Multivariate analysis did not show a difference between autologous and allogeneic in concern to relapse/progression, but NRM was higher in the allogeneic group and in patients with two or more lines of pretransplantation chemotherapy. Patients who underwent autoHCT in CR1 showed the highest survival rates. Moreover, patients presenting ALCL subtype had better survival rate (55% vs 35% PFS and 68% vs 41% OS) and reduced NRM in the autoHCT setting in comparison to alloHCT. This is the largest retrospective study regarding alloHCT in PTCL, with 126 patients undergoing this modality of transplantation, and they found that patients not in CR or after two or more chemotherapy regimens were at higher risk of overall mortality and treatment failure. Also, they described no impact of donor source, conditioning intensity, or graft-versus-host disease (GvHD) on relapse or survival (19).

A systematic review and meta-analysis published in 2021 was designed with the aim of comparing the efficacy and safety of auto and alloHCT in patients with R/R PTCL. Thirty studies were analyzed, comprising a total of 1765 patients. The rate of 3-year OS was relatively higher on the auto group (55% vs 50%, in comparison to allo), although CR rate prior to transplant was higher on patients who underwent autologous transplant, indicating that these patients were more chemosensitive. Therefore, when taking this enrollment bias into consideration, it is possible to speculate that alloHCT showed survival advantage in comparison to autoHCT. However, 3-year TRM was lower on the autologous group (7% vs 32%), showing that despite providing greater effectiveness, alloHCT may be riskier than autoHCT (42).

As presented in the articles reviewed, the evidence to support the use of autologous and allogeneic HCT in the R/R PTCL are limited and conflicting, meaning this is still a challenging scenario for physicians. Additional prospective trials and new therapeutic approaches, including cell therapy techniques, are sorely needed in this population.

## 4 Advanced cellular therapies for PTCL

CAR technology enables the cytotoxic immune cells to specifically recognize and target a surface antigen in a major histocompatibility complex (MHC)-independent manner. The CAR extracellular single-chain variable fragment (scFv) is specific for the target antigen and is linked by hinge and transmembrane regions to CD28 and/or 4-1BB co-activation domains and finally the CD3ζ intracellular signaling domain (43). Developing a safe and effective CAR-T cell therapy relies on identifying an ideal surface target antigen that is highly sensitive for the underlying malignancy and uniformly specific to avoid on-target off-tumor toxicities. Hematological malignancies are generally heterogeneous, and an optimal antigen that is exclusively expressed on all malignant cells with robust intensity is rarely found. Although not as studied as CD19+ leukemia and lymphomas and other hematological malignancies, the number of CAR-T cell therapy clinical trials for T cell malignancies have been increasing considerably in the past few years (**Table 3**).

CAR transgenes can be introduced into cells either transiently using mRNA electroporation or permanently using lentiviral, gammaretroviral or transposon-based gene delivery (44, 45). CAR-T products generated through viral vector transduction can lead to robust expansion and persistence *in vivo*, which heightens the risk of T-cell aplasia(46). Conversely, mRNA-engineered CAR-T cells have demonstrated similar anti-tumor activity but with limited persistence following administration (47, 48). This strategy shows promise for treating T-cell malignancies, but stable and sufficient tumoricidal activity may require sequential CAR-T administration or bridging to HCT. To address T-cell aplasia, equipping CAR-T products with safety switches (or suicide switches) may allow the control of transduced T-cells after infusion into patients (49).

Due to the similar biological structures and functions shared by B- and T-cells, chimeric antigen receptor (CAR) T-cell therapy was initially considered a natural approach for treating T-cell neoplasms. However, practical concerns such as fratricide and possibility of immunosuppression due to aplasia of normal T-cells have been raised. These concerns are further complicated by an immunosuppressive microenvironment that promotes the development and progression of T-cell malignancies, particularly TCLs (50, 51). Moreover, autologous T-cells harvested from patients with T-cell malignancies for CAR-T cell manufacture may be contaminated with malignant cells. To circumvent that issue, the use of allogeneic T-cells, Natural Killer (NK) cells, iNKT cells and macrophages are currently being explored. Indeed, several studies have evaluated these different cell types as allogeneic “off-the-shelf” products. In the following sections we present recent studies and discuss its advantages.

## 5 CAR-T cell for PTCL

**Figure 1:**
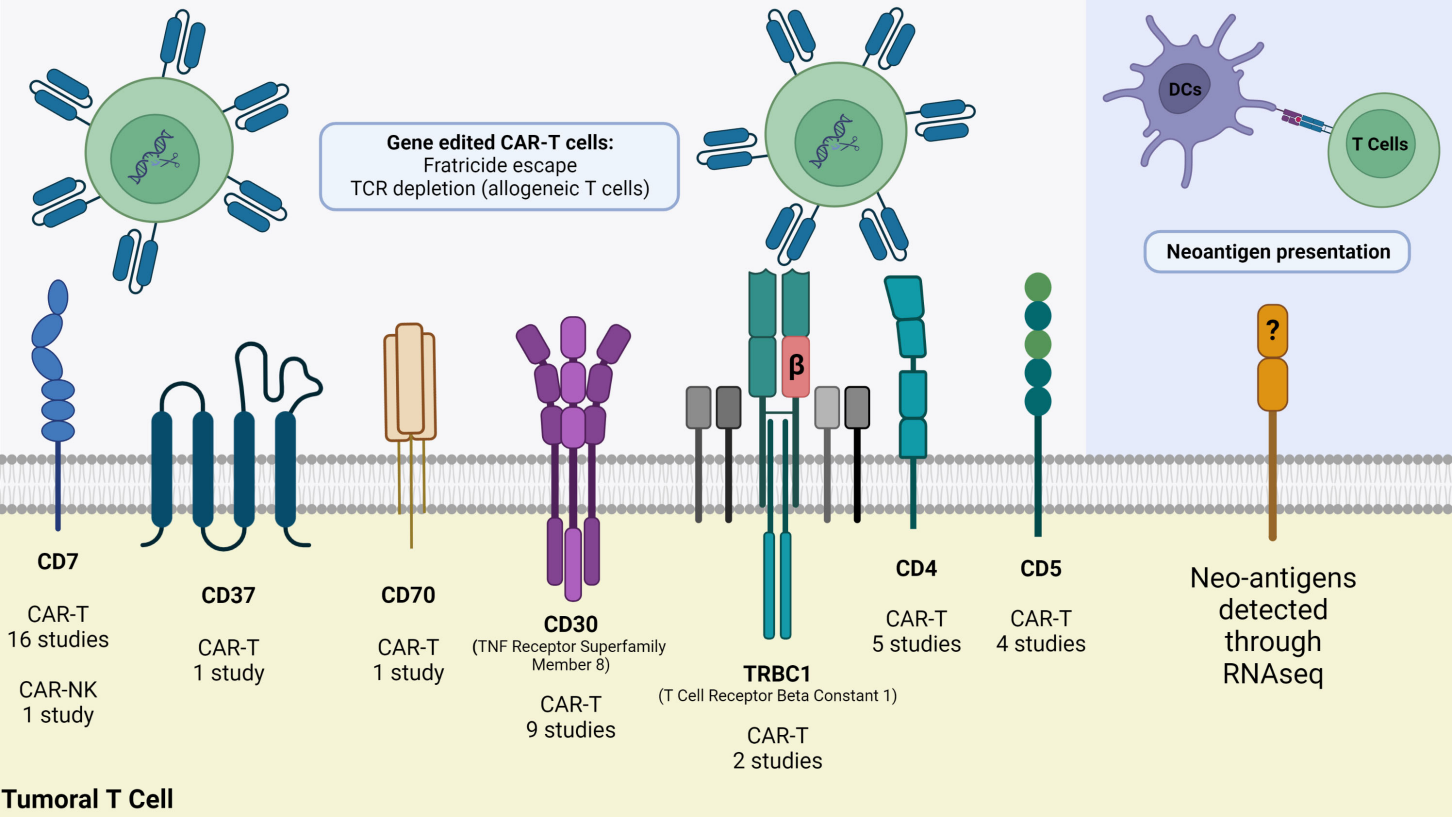
Strategies to fight PTCL using advanced cellular therapies. At least seven T cell membrane markers are being currently targeted in clinical trials, but CAR-T cells demand gene editing whether to avoid fratricide (CAR-T/NK cells self-destruction) or Graft-versus-Host-Disease (GvHD) when using allogeneic T cells. As an alternative strategy, RNA-seq of malignant T cells reveal neoantigens that can be synthesized and delivered to dendritic cells for presentation through MHC molecules to T cells. Image was created using Biorender.

Most targeted antigens for CAR-T products against T-cell malignancies, such as CD3, CD5, and CD7, are commonly expressed by healthy T-cells (52, 53). This shared expression makes it difficult to isolate healthy T-cells from patients with T-cell malignancies to engineer autologous CAR-T products, where normal and malignant T-cells might be collected during leukapheresis. A CAR-T construct targeting a tumor-associated antigen (TAA) expressed by different populations of T-cells may present on-target off-tumor effect, attacking and destroying malignant and normal T-cells, as well as other CAR T-cells, leading to disruption of CAR-T cells’ expansion, persistence, and tumoricidal function. Such fratricide could result in T cell dysfunction before or after exposure, leading to resistance to CAR-T therapy or disease relapse (54, 55). These observations prompted several groups to develop various products and strategies, including targeting more restricted T-cell antigens, such as CD4, CD30, CD37, and CCR4. Selecting the appropriate target and considering potential adverse events remains a challenge in CAR-T therapy for T-cell malignancies. Other alternative antigens, such as the myeloid markers CD13 and CD33, are emerging as possible targets due to their aberrant expression on precursor T-cell leukemia, which could indicate a worse disease prognosis (56). A quick search in the ClinicalTrials.gov repository using the terms “Peripheral T cell lymphoma”, “PTCL”, “T cell Lymphoma”, “CAR” and “chimeric antigen receptor” returned 40 studies of which 39 are CAR-T cell studies and only one is a CAR-NK clinical trial. It is possible to note from these studies that several T cell membrane markers are being investigated as possible targets to CAR-T cell therapy for PTCL (**Figure 1**). The most applied target is CD7, a protein of the immunoglobulin superfamily expressed both in mature T cells and thymocytes. Currently, eight different markers are being tested as targets for CAR therapy for PTCL: CD4, CD5, CD7, CD30, CD37, CD70, CD147 and TRBC1 (**Table 3**).

**Table 3:** Registered clinical trials using CAR-T/NK cells to treat PTCL patients.

Target	Number of Studies	Source	Trial Number	Status	Phase	N	Start Year
**CD4**	5	Auto (5)	NCT04162340	Recruiting	1	12	2019
			NCT03829540	Active, not recruiting	1	20	2019
			NCT04712864	Active, not recruiting	1	50	2021
			NCT04973527	Terminated	1	9	2021
			NCT04219319	Terminated	1	4	2021
**CD5**	4	Auto (3)	NCT03081910	Recruiting	1	42	2017
			NCT04767308	Not yet recruiting	Early 1	18	2021
			NCT05138458	Recruiting	1/2	40	2021
		Unknown (1)	NCT04594135	Recruiting	1	20	2020
**CD7**	17	Allo (6)	NCT02742727	Recruiting	1	21	2016
			NCT04538599	Completed	1	12	2020
			NCT05127135	Recruiting	1	24	2020
			NCT04689659	Recruiting	2	50	2021
			NCT04264078	Recruiting	2	20	2021
			NCT05377827	Recruiting	1	20	2022
		Auto (5)	NCT04004637	Not yet recruiting	1	48	2019
			NCT04480788	Recruiting	1	9	2020
			NCT05059912	Recruiting	Early 1	30	2021
			NCT04840875	Recruiting	1	30	2021
			NCT03690011	Recruiting	1	20	2021
		Unknown (6)	NCT04572308	Completed	1	20	2020
			NCT04934774	Recruiting	1	4	2020
			NCT04823091	Recruiting	1	20	2021
			NCT05620680	Unknown status	1/2	10	2022
			ISRCTN15323014	Recruiting	1	10	2022
			NCT05290155	Unknown status	1	10	2022
**CD30**	9	Auto (7)	NCT03049449	Completed (Has results)	1	26	2017
			NCT03602157	Recruiting	1	59	2018
			NCT04008394	Recruiting	1	50	2019
			NCT04083495	Recruiting	2	20	2019
			NCT04653649	Recruiting	1/2	30	2020
			NCT04526834	Active, not recruiting	1	21	2021
			NCT05208853	Not yet recruiting	Early 1	9	2022
		Allo (2)	NCT04288726	Recruiting	1	18	2020
			NCT04952584	Not yet recruiting	1	18	2023
**CD37**	1	Auto	NCT04136275	Recruiting	1	18	2020
**CD70**	1	Allo	NCT04502446	Recruiting	1	45	2020
**CD147**	1	Unknown	NCT05013372	Not yet recruiting	Early 1	12	2023
**TRBC1**	2	Auto (1)	NCT03590574	Recruiting	1/2	200	2018
		Unknown (1)	NCT04828174	Recruiting	1	9	2021

Even though malignant T-cells significantly decrease the expression of T cell receptor (TCR), this important receptor is still expressed in about 30% of T-ALLs and the vast majority of PTCLs (57). Since a normal population of T cells expresses both TRBC1 and TRBC2, and a malignant subset expresses only one, targeting either receptor individually could have antitumor effects while preserving a substantial portion of normal T-cells. *In vitro* studies on the generation of TRBC1CAR-T have demonstrated the ability to leave TRBC2+ cells untouched. In addition, treatment with these cells was associated with a significant decrease in tumor burden and prolonged survival in comparison to the control group (58). This led to the use of CARs specific for the TCR beta chain constant regions TCRB1 or TRCB2, which is being evaluated in a phase I/II clinical trial for T-non-Hodgkin lymphomas (NCT03590574). Malignant Sezary cells express a unique TCR gene rearrangement, which could be a good tumor-specific antigen to be targeted. However, this unique TCR seems to vary from patient to patient, making TCR targeting a complex approach (59). Certain highly expressed cell surface molecules such as CCR4, programmed cell death protein 1 (PD-1), and CD47 could provide a common targeting approach. CD47 is significantly increased on Sezary cells and inhibits phagocytosis by macrophages (60). Studies showed that targeting of universal T cell antigens such as CD3 and CD7 using CAR-T cells leads to fratricide of CAR-T cells. Other tumor-restricted antigens targeted with CAR-T cells include CCR4, CD4, and CD30. PTCLs are frequently characterized by the presence of CD4+ cells, with consistent and elevated expression levels of CD4 making it an optimal candidate for targeted therapy using CAR technology (61). Targeting of CD4 led to fratricide of CD4+ CAR-T cells, but the remaining CD8+ CAR-T cells may have therapeutic potential (62).

In 2021, Pan et al. (63) reported the first-in-human Phase I trial to evaluate the safety and efficacy of donor-derived CD7 CAR T-cell therapy for patients with R/R T-ALL. Twenty patients diagnosed with CD7-positive r/r T-ALL were enrolled. The median age of the participants was 11 years (range, 2-43), and they had received at least two previous therapies. To address the issue of CD7 CAR T-cell fratricide, the researchers designed a CAR construct using IntraBlock technology, which effectively prevented CD7 cell surface expression. To overcome the challenge of low quantity and quality of patients’ autologous T cells, CAR-T cells were manufactured using T cells harvested from previous SCT donors and new donors. Most patients experienced Grade 1-2 CRS, with only 10% of patients experiencing Grade 3 CRS. Neurotoxicities associated with the treatment were mild and self-limiting. The treatment demonstrated a high response rate, with 95% of the participants responding to the therapy. Furthermore, 90% of the patients achieved complete remission, including 85% who achieved MRD-negative CR by day 15. Within 15 days post-infusion, the therapy led to a rapid depletion of CD7-positive normal lymphocytes, including T cells. However, CD7-negative T cells dramatically increased in all patients, indicating a gradual recovery of total T cells and NK cells. These T cells also displayed lower T-cell receptor diversity. Nevertheless, they demonstrated the ability to react to fungal and viral stimulations, producing interferon-gamma, suggesting potential immune-protecting functions. The trial demonstrated an acceptable safety profile, with most adverse events occurring within 30 days post-infusion. Early GVHD was observed in 60% of the participants but was low grade and manageable. CAR T cells efficiently proliferated and persisted in patients without evidence of rejection, which may be attributed to complete chimerism in those who received donor-derived cells after previous stem cell transplantation. A multi-center, phase II study is currently in progress to investigate the use of donor-derived CD7 CAR-T cells in patients with R/R T-cell malignancies (63).

More recently, Tan et al. reported the outcomes of the same cohort described in 63, after a median follow-up time of 27 months post-treatment. Non-relapse mortality occurred in 25% of the 20 patients, with a median time of 6.8 months after treatment. The causes of non-relapse mortality included infections in patients without SCT consolidation and engraftment syndrome in a patient after SCT consolidation. Of the 19 patients who responded, seven proceeded to SCT consolidation, two withdrew to take alternative therapies, and ten did not receive further therapy. Among the ten patients who did not receive further treatment, three were in remission, three relapsed (including one with CD7-positive extramedullary disease), and four died of infection. Among the seven patients who underwent SCT consolidation, three maintained in remission, three relapsed, and one patient died of transplant-related complications. The relapse rate among responders was 33.3%, with a median time to relapse of 6 months post-infusion. The median PFS and duration of response (DOR) were 11.0 months and 10.5 months, respectively, among the 19 responders. The median OS was 18.3 months. The 2-year PFS rate for responders and OS rate for all 20 treated patients were 36.8% and 42.3%, respectively. Subgroup analysis revealed that patients without SCT consolidation had a 2-year PFS rate of 31.8% and an OS rate of 35%, with a median PFS and OS of 11.0 months and 18.3 months, respectively. In contrast, patients with SCT consolidation had a 2-year PFS rate of 42.9% and an OS rate of 58%, with a median PFS of 9.1 months. The study did not reach the median OS for patients who received SCT consolidation. Long-term monitoring of T-cell phenotype and function showed that CD7+ T and NK cells remained undetectable in all patients until the last visit, except for one patient who experienced recovery of CD7+ T and NK cells 25.6 months post-infusion following the loss of flow-cytometry-detectable CD7 CAR T cells at 22.7 months. The number of CD7− T, total T, and NK cells progressively increased, with total T-cell counts recovering to normal levels in 7 out of 12 patients at a median time of 1.9 months. Furthermore, long-term monitoring of T-cell phenotype and function in two patients revealed that the central memory T-cell subpopulation gradually increased, while low levels of naive and stem-cell memory T-cell subpopulations were detectable in one patient after 15 months. TCR diversity in patients after CD7 CAR T-cell infusion remained lower compared to healthy donors. The study identified late-onset GVHD as the most common long-term adverse event, with an incidence of 58% among the 12 patients without SCT consolidation. The authors suggest that early bridging to SCT consolidation may reduce the risk of severe infections, which were observed in a lower incidence (14.3%) in patients with SCT consolidation after CAR T-cell infusion. Relapse analysis revealed that among the patients who relapsed, four had CD7-negative relapses, and two had CD7-positive relapse. Frameshift or missense mutations were detected in the four CD7-negative relapse patients, suggesting that mutations may be a main cause of CD7 loss in tumor cells. The study also observed CD7-positive relapse following the loss of CAR T cells in a patient without SCT consolidation, suggesting that insufficient persistence of CAR T cells may contribute to relapse. The study acknowledges several limitations, including its phase I trial design with a small sample size. Consequently, the authors emphasize the need for larger phase II studies to confirm the safety, efficacy, and prognostic factors associated with CD7 CAR T-cell therapy. Despite these limitations, the study’s strength lies in its provision of the first long-term follow-up in patients with T-ALL after CAR T-cell treatment. The durable responses observed, along with new signals of long-term adverse events, support the feasibility of using donor-derived CD7 CAR T cells as salvage treatment for children or adults with R/R T-ALL. Notably, severe infection emerged as a significant side effect associated with this therapy, underscoring the importance of early SCT consolidation and vigilant monitoring and prevention of infections for patients for whom subsequent SCT is not feasible (64).

Zhang et al. have recently presented the outcomes of a single-center phase I study evaluating autologous and allogeneic anti-CD7 CAR-T cell therapy in patients with R/R T-cell malignancies. The trial enrolled 11 patients aged 16 to 69, with CD7+ T-cell malignancies, including adult T-ALL, T-cell lymphoblastic lymphoma, angioimmunoblastic T-cell lymphoma, and mycosis fungoides. Of these patients, 10 received treatment, after one patient achieved complete remission prior to CAR-T cell infusion. The median lines of previous therapies were 4. CAR-T cells were produced using PBMCs from either patients or donors. The authors observed that 70% of patients achieved CR with mild CRS and no ICANS. Patients who received allogeneic CAR-T cells did not experience severe CRS, ICANS, or GVHD. In addition to these common complications, hematological toxicities, hemophagocytic lymphohistiocytosis (HLH), infections, and T-cell aplasia were also observed. The choice of allogeneic CAR-T cells for patients with highly aggressive and rapidly progressing malignancies eliminated the need for a one-month drug elution period before leukapheresis, reducing the likelihood of rapid disease progression. The CR rate was 80% for patients receiving allogeneic cells compared to 40% for those with autologous products. Notably, the relapse rate showed significant differences, with only one patient (25%) experiencing CD7- recurrence after allogeneic CAR-T cell therapy, while all patients treated with autologous CAR-T cells relapsed, either in the bone marrow or as extramedullary disease. Some patients experienced CD7+ recurrence despite the absence of detectable CAR copies *in vivo*. The study highlighted that the lower persistence of autologous CAR-T cells may contribute to treatment failure. Allogeneic CAR-T cells demonstrated stable survival in 75% of patients at month 2, whereas only 33% of patients receiving autologous cells showed persistent CAR-T cell presence. Two patients with Epstein-Barr virus (EBV) activation died of pneumonia during the study, emphasizing the need for caution when enrolling patients with a history of EBV infection and the importance of close monitoring. Limitations of the study include its non-randomized controlled trial design due to patient conditions and the small sample size. The authors emphasized the need for extended follow-up and larger studies to further evaluate long-term outcomes of anti-CD7 CAR-T cell therapies in T-cell malignancies (65).

Using a different approach to avoid fratricide during anti-CD7 CAR-T manufacture, Lu et al. showed the development of naturally selected CD7 CAR T cells (NS7CAR) for the treatment of CD7+ T-cell malignancies, focusing on T-ALL/LBL. The authors employed lentiviral transduction and selection techniques to generate NS7CAR T cells that retain CD7 expression while avoiding self-targeting and fratricide. A first-in-human phase 1 clinical study was conducted, utilizing NS7CAR T cells derived from patients with treatment-refractory T-ALL/LBL or their hematopoietic stem cell donors. Twenty patients, aged 3 to 47 years, received NS7CAR T cell therapy. Most patients experienced mild CRS, with only one patient developing grade 3 CRS, and neurotoxicity was minimal. At the 28-day evaluation, 19 patients achieved measurable residual disease-negative complete remission or incomplete CR, including responses in patients with extramedullary disease. Subsequent transplantation was performed in most patients, and no relapses were documented during a median follow-up of 142.5 days. Importantly, NS7CAR T cell therapy resulted in rapid ablation of circulating CD7+ T cells and natural killer (NK) cells, which were replenished by CD7-negative subsets, preventing prolonged T-cell and NK-cell aplasia. The study concluded that NS7CAR T cells are well-tolerated and effective against CD7+ T-cell malignancies. Importantly, the generation of naturally fratricide-resistant CD7 CAR-T cells simplifies the manufacturing process and enhances the cost-effectiveness of this therapy (66).

## 6 “Universal” CAR-T cell for T-cell malignancies

Gene-editing methods have been gaining prominence in the cellular therapy field, such as the transcription activator-like effector nuclease (TALEN), which is being used to disrupt the CD3/TCR complex and prevent the expression of endogenous TCR in T-cells. Once TCR is inhibited, the cells are then modified to express CD3ϵ-targeting CARs. This approach has shown impressive results, with specific and significant antitumor activity against pediatric T-ALL samples demonstrated in preclinical models with the CD3+ Jurkat cell line (67). In a separate study, gene editing technologies were employed to simultaneously remove the expression of one of the TRBC genes, resulting in the elimination of endogenous TCR from the cell surface, indicating that this strategy could be used to prevent fratricide when producing autologous TRBCCAR-T (68). Other gene-editing techniques, such as CRISPR-Cas9, and Zinc-finger nucleases (ZFN), have been investigated for their potential to develop off-the-shelf CAR-T products for T-cell neoplasms. CRISPR-Cas9 has also been used to knock-out CD5 in T cells before embedding the CAR transgene into primary patient cells and Jurkat cells (69). This approach has resulted in limited fratricide and subsequent CAR persistence. CRISPR-Cas9 genome editing to disrupt the CD7 expression and engineer CAR-T cells lacking CD7 and TCR alpha chain (TRAC) expression have demonstrated significant antitumor activity against T-ALL cell lines and primary human samples, as well as tumor regression in preclinical models with the absence of GvHD (55).

Hu et al. presented the results of a phase I clinical study investigating the safety, efficacy, and pharmacokinetics of genetically modified CD7-targeting allogeneic CAR-T cell therapy (RD13-01) in patients with R/R CD7-positive hematological malignancies. The RD13-01 CAR-T cells were designed to enhance persistence and potency by genetically depleting CD7, TCR, and HLA class II, and incorporating an NK cell inhibitor (NKi) and the common cytokine receptor γ chain (γc). Preclinical assessments demonstrated potent antitumor activity of RD13-01 CAR-T cells. In the clinical trial, RD13-01 CAR-T cells were manufactured from allogeneic healthy donor PBMCs, and residual TCR/CD3+ T cells were removed to minimize GvHD. Twelve eligible patients with relapsed or refractory hematological malignancies were enrolled, including T-ALL, T-cell lymphoma, and acute myeloid leukemia (AML) cases. No dose-limiting toxicity, GvHD, or ICANS occurred during the trial. Grade 1-2 CRS was observed in 10 patients, with no severe CRS (grade ≥3) reported. Among the 11 patients evaluated for efficacy, 82% achieved an objective response, with 64% achieving complete remission or CR with incomplete hematological recovery at day 28 post-infusion. At a median follow-up of 10.5 months, four responders remained in CR, while one patient underwent salvage HCT and remained in CR. However, relapse or disease progression occurred in three leukemia and one lymphoma patient at a median time of 82 days after infusion. The expansion of CD8+CD7− T cells, which may recognize the HLA antigen of infused CAR-T cells, was associated with decreased CAR-T cell numbers and antigen-positive relapse. On the other hand, however, as 63 have shown, CD7− T cells generated from CD7-depleted hematopoietic stem cells may contribute to maintaining T cell function and controlling infections. Strategies to prolong CAR-T cell persistence while preserving CD7− normal T cell expansion, such as the expression of inhibitory ligands (e.g., PD-L1) on allogeneic CAR-T cells, warrant further investigation. Additionally, the authors report inter-patient variability in clinical responses following infusion of cells manufactured from the same batch, suggesting that endogenous factors and recipient immune landscape may influence therapeutic outcomes in the context of a universal CAR-T therapy (70).

In a more recent work, 71 demonstrated the use of base editing, mediated by CRISPR technology, for precise DNA modifications without inducing double-stranded DNA breaks, to generate base-edited allogeneic CAR7 (BE-CAR7) T cells for a phase I feasibility and safety trial in pediatric patients with R/R T-ALL. Healthy donor T cells were electroporated with specific single-guiding RNAs (sgRNAs) and a codon-optimized cytidine base editor (coBE) mRNA to target TRBC1, TRBC2, CD7, and CD52 genes. The edited T cells were then transduced with a lentivirus vector encoding a CAR targeting CD7. The trial aims to recruit 10 children in the United Kingdom for an initial cohort. The initial report covers data from lympho-depletion to day 28 after CAR-T cell infusion for three patients. Patients in molecular remission at day 28 underwent allogeneic stem-cell transplantation, depleting any persisting BE-CAR7 cells through the conditioning regimen before the transplant. Molecular analysis confirmed the precise editing of targeted cytosine positions in TRBC, CD7, and CD52. Karyotyping showed normal karyotypes, and PCR assays had negative results for translocations. The 28-day treatment period led to significant antileukemic responses and deep remission in two of the three patients. Cytokine release syndrome (CRS), fever, rash, and multilineage cytopenia were observed in all patients, with infectious complications managed with antiviral medications. The authors acknowledge the substantial immunosuppressive and cytopenic effects of the protocol and the risks associated with immune-cell manipulation. Subsequent allogeneic transplantation was performed to ensure immune reconstitution and limit the persistence of engineered cells (71).

## 7 CAR-NK cell for PTCL

Prior pre-clinical investigations have employed CAR-modified primary human natural killer (NK) cells against CD19, CD20, CD244, and HER2 for both hematological and solid tumors (72–75). Additionally, clinical trials have demonstrated successful application of anti-CD19 CAR-modified umbilical cord blood-derived and haploidentical NK cells in patients with CD19 lymphoid tumors and acute myeloid leukemia, respectively (76, 77). The use of CAR-modified NK cells can potentially eliminate the risk of fratricide, T-cell aplasia, and GvHD associated with CAR-T therapy (78). It may also remove the need for an inducible safety switch, as CAR-modified NK cells are eliminated shortly after administration (79). In contrast to CAR T-cells, CAR NK cells offer the benefit of engaging tumor cells through diverse mechanisms, while exhibiting a relatively reduced production of pro-inflammatory cytokines (80, 81).

The human NK cell line NK-92 has been utilized in multiple clinical studies for both hematologic malignancies and solid tumors, as well as in pre-clinical CAR applications (82–84). Given its versatility in both clonal NK cells and autologous/allogeneic NK cell immunotherapy, NK-92 serves as a valuable model. A third-generation CD5-CAR incorporating NK-92 cell lines, which do not express CD5 on their surface, showed selective and significant tumoricidal activity towards various T-cell lines, including Jurkat, CCRF-CEM, and MOLT-4, as well as against primary CD5+ cells from human T-ALL and PTCL samples (85). To mitigate the risk of T-cell aplasia and related infections, researchers engineered a CD4-redirected CAR-NK using the NK-92 cell line (61). Ex vivo experiments have shown that CD4CAR NK-92 cells possess potent anti-tumor cytotoxicity against various adult and pediatric CD4+ lymphoma/leukemia cell lines, as well as primary CD4+ T-cell malignancies from both adult and pediatric patients. In xenogeneic mouse models, CD4CAR NK-92 cells also demonstrated strong *in vivo* anti-CD4 activity. Notably, CD4CAR NK-92 cells did not affect the CFU capacity of CD34+ cord blood granulocyte/macrophage or erythroid cells in ex vivo assays, indicating that they do not compromise the hematopoietic stem cell and progenitor compartment. This promising approach suggests that CD4CAR NK cells could be utilized as part of a bridge-to-transplant strategy or as a stand-alone curative treatment for patients who are not eligible for HCT. Moreover, CD3CAR transduced NK-92 cells showed significant dose-dependent *in vitro* and *in vivo* cytotoxic against CD3-expressing PTCL samples and several T-ALL cell lines, with prolonged survival in preclinical models engrafted with the Jurkat cell line (86). In a recent study, CAR-NKs with the 2B4 and 4-1BB costimulatory domains demonstrated similar selective tumoricidal activity *in vitro*, while CAR-NKs with the 2B4 co-stimulatory domain showed an improved antileukemic activity in T-ALL preclinical models (87).

## 8 Future perspectives

CAR-T cell therapy has faced challenges in achieving optimal trafficking to challenging tumor sites, such as the skin. This is thought to be due to poor infiltration of such areas by αβ T cell subsets. γδ T cells, which constitute a smaller population of circulating lymphocytes (1-5%), are present in the skin, intestine, and reproductive organs and express chemokine receptors that attract them to these inaccessible tumor locations (88, 89). Additionally, γδ T cells can proliferate ex vivo and do not induce GvHD due to MHC-independent activation of their TCR. Therefore, γδ T cells could be considered as potential alternative effectors for allogeneic CAR-T therapy in T-cell malignancies, following thorough evaluation in studies for other malignancies (90, 91). Multi-virus-specific T (VST) cells have also been utilized as effector cells for CAR expression. Such cells can be genetically engineered to lack CAR target antigen expression and be fratricide-resistant, offering potential antiviral activity in the case of T-cell aplasia (92, 93). Studies have shown that allogeneic VST cells with HLA alloreactivity do not cause GvHD in humans, suggesting they may provide an alternative for producing off-the-shelf CAR-Ts (94).

The potential of CAR-iNKT (Invariant Natural Killer T) cell therapy as an “off-the-shelf” allogeneic immunotherapy for the treatment of T cell lymphoma is also being investigated. iNKT cells are a rare subset of T cells with innate and adaptive immune features (95). They express an invariant TCRVa24Ja18 chain that pairs with diverse TCRVb11 chains (96). By targeting specific TCRVb chains associated with ATL/TCL, CAR-iNKT cells can be engineered for effective and precise immunotherapy. In a very elegant pre-clinical study, (97) generated lentiviral CAR constructs to target TCRVb1, Vb2, Vb9, and Vb11 expressed on T cells. CAR-iNKT cells engineered with these constructs demonstrated potent anti-tumor activity against primary ATL cancer cells. TCRVb2 CAR-iNKT cells significantly inhibited tumor growth without causing adverse effects such as weight loss or signs of acute GVHD in animal models. The findings suggest that anti-TCRVb CAR-iNKT cells can offer both autologous and allogeneic treatment options as “off-the-shelf” cellular immunotherapy for T cell malignancies. Early clinical experience with allogeneic CAR-iNKT cells against B cell lymphoma have already indicated minimal toxicity and aGVHD (98). Overall, this study provides a promising rationale for the clinical development of anti-TCRVb CAR-iNKT cells as an effective and highly selective immunotherapy for currently incurable T cell lymphomas.

Another interesting approach to target PTCL that has already been evaluated in clinical studies is based on the use of neoantigen-activated haploidentical T cell therapy (NAHTC) (99). Whole-exome sequencing was used to identify non-synonymous mutations in matched tumor and normal cells, as well as tumor RNA sequencing to identify neoantigen candidate epitope sequences. The predicted binding affinity of peptides to individual HLA molecules was then determined, and synthetic RNAs encoding potential neo-epitopes were designed based on the selected mutations. Haploidentical donor’s monocytes were isolated and differentiated into mature dendritic cells (DCs) that were electroporated with selected synthetic RNA and then co-cultured with haploidentical T cells. Preliminary findings demonstrate that the NAHTC regimen was well-tolerated, with no incidence of CRS or GvHD in the treated patients. Of the five PTCL patients evaluated, four (80%) achieved a complete response, and three of the four CRs were sustained until the last analysis. Thus, treatment with NAHTC was safe and resulted in durable clinical responses. Despite the small sample size and preliminary nature of these findings, single-dose NAHTC therapy demonstrated superior activity compared to recently FDA-approved drugs for R/R PTCL (100–102). The effectiveness of NAHTC therapy for PTCL may stem from its distinct mechanism of action. Tumor neoantigens represent *de novo* epitopes derived from somatic mutations and are therefore tumor-specific and highly immunogenic, as they lack central tolerance. NAHTC cells activated by multiple patient-specific neoantigens *in vitro* are likely to target a diverse array of malignant clones within each patient, with the potential to address tumor heterogeneity, reducing the likelihood of tumor escape by single neoantigen loss (103, 104). The use of T cells from healthy donors in haploidentical T-cell therapy ensures their manufacture quality and quantity, making it a simple, safe, and reliable process that saves time and reduces costs, ensuring the accessibility of this treatment to more patients.

## 9 Conclusion

Autologous HCT has been the core of consolidation therapy in most of the more common PTCL subtypes for chemoresponsive patients (2, 35). Only recently, after the first randomized phase III ECHELON-2 study, there is evidence of improvement in PFS and OS in comparison to standard induction regimen CHOP (105). The expression of ALK in ALCL correlates with a favorable prognosis compared with other histological subtypes of PTCL, therefore, autoHCT may be considered in high-risk IPI patients or in second-line therapy. On the other hand, acute and lymphomatous subtypes of ATLL have a poor prognosis, with recommendation of alloHCT even in first-line therapy (2, 35). Overall, there is limited data and a lack of randomized trials for PTCL treatment, mainly due to the low incidence and heterogeneity of histology subtypes, and therefore, current treatments rely mostly on phase II trials, retrospective studies, and expert opinion (106). Overall, it is possible to conclude that HCT is a reasonable strategy for PTCL patients in the R/R context, but it seems that main factors to take into consideration may be the type of transplant (auto/allo) and the stage of the disease to be performed. Currently, HCT is the only potentially curative therapy for patients with R/R PTCL, and the evidence for the role of auto and alloHCT for this population has only been evaluated in registry data and retrospective studies (4). It is noteworthy that conclusions were not homogeneous among authors. In general, it might be safe to assume that autoHCT often results in lower durable benefit for patients with R/R disease in comparison to alloHCT, however, for patients with ALCL subtype and chemosensitive disease, autoHCT may provide survival benefit. These findings corroborate with the current recommendations from the American Society for Blood and Marrow Transplantation, that recommends autoHCT for nodal PTCL subtypes with relapsed chemosensitive disease, if it has not been done as upfront consolidation, or alloHCT when an autologous transplantation was made in first line (35). CAR-T cell therapy is currently being considered as a possible approach for the treatment of PTCL, but challenges like fratricide, failure in manufacture and proper antigen targeting still need to be addressed. Clinical studies that use allogeneic anti-CD7 CAR-T have shown promising results, indicating it as an effective “bridge-to-transplant” strategy for patients with available hematopoietic cell donors. Novel approaches such as the use of other immune cells, like NK and myeloid cells equipped with a CAR as “off-the-shelf” products are being studied, as well as the modification of donor derived T cells through gene editing techniques (107). With the rapid evolving knowledge of PTCL molecular and pathogenic properties, we will be able to develop efficient and personalized therapies for the treatment of these hard-to-treat diseases.

## Author contributions

SC, AK, CA, TO, PK and VR reviewed the literature, organized, and wrote the manuscript. All authors contributed to the article and approved the submitted version.
